# Interpretable non-invasive glucose monitoring: an attention-based deep learning framework for visualizing hemodynamic correlates in PPG signals

**DOI:** 10.3389/fbioe.2026.1843361

**Published:** 2026-07-03

**Authors:** Rizal Syamsul, Kim Dong-Seong

**Affiliations:** 1 ICT-CRC, Kumoh National Institute of Technology, Gumi, Republic of Korea; 2 School of Electrical Engineering, Telkom University, Bandung, Indonesia; 3 NSLab Co. Ltd, Gumi, Republic of Korea

**Keywords:** blood glucose estimation, deep learning, gated recurrent unit (GRU), long short-term memory (LSTM), noninvasive monitoring, photoplethysmography (PPG), recurrent neural networks (RNN)

## Abstract

**Background:**

Diabetes mellitus necessitates frequent blood glucose monitoring, yet invasive finger-prick methods remain a barrier to adherence. Although Photoplethysmography (PPG) offers a promising noninvasive alternative, current deep learning approaches largely operate as “black boxes,” obscuring the physiological features driving their predictions and limiting clinical trust.

**Methods:**

To address this interpretability gap, we propose a novel Attention - Guided Convolutional - Recurrent Neural Network (AG-CRNN). Unlike standard deep learning models that treat the entire signal uniformly, our architecture integrates a Temporal Attention Mechanism that dynamically assigns importance weights to specific segments of the PPG waveform. This allows the model to learn and emphasize hemodynamic fluctuations associated with glucose variability, while suppressing motion artifacts. We validated the framework using a dataset of synchronized PPG and glucose measurements from 23 subjects.

**Results:**

The proposed attention-based model achieved a Mean Absolute Error (MAE) of 11.59 mg/dL, significantly outperforming standard LSTM and GRU baselines. Crucially, our Explainable AI (XAI) analysis revealed that the model preferentially focuses on the *systolic decay* and *diastolic decay* regions. This visualization aligns with the established physiological evidence linking hyperglycemia to vascular stiffness and wave reflection timing.

**Conclusion:**

This study presents a high-accuracy glucose estimation framework that provides visual explanations of its decision-making process. By demonstrating that the model leverages genuine morphological correlates rather than spurious noise, we bridged the gap between AI performance and clinical plausibility.

## 1 Introduction

Diabetes mellitus (DM) represents a significant global health challenge, contributing to an elevated cardiovascular risk, diminished quality of life, and considerable economic burden. Effective management is contingent on the regular monitoring of blood glucose (BG) levels to inform insulin dosing and prevent complications Sun et al. (2024); Cappon et al. (2019). While invasive finger-prick tests and continuous glucose monitoring (CGM) systems are the current standards of care, their invasiveness and cost often hinder long-term adherence. Consequently, there is a critical demand for noninvasive monitoring solutions that utilize ubiquitous wearable sensors.

Photoplethysmography (PPG), which measures volumetric blood changes in peripheral vessels, has emerged as a promising candidate for this purpose Jiang et al. (2025). From a physiological standpoint, blood glucose levels modulate vascular tone and endothelial functions. Acute hyperglycemia is linked to increased blood viscosity and arterial stiffness, which subtly alters the morphology of the pulse wave, particularly in the reflected wave components Rodriguez-León et al. (2021); Satter et al. (2024); Rizal and Kim (2025). These hemodynamic changes suggest that the PPG waveform contains latent information correlated with the glucose levels.

However, extracting this information is nontrivial because of signal noise and intersubject variability. Historically, researchers have relied on manual feature engineering and linear regression Yi et al. (2019); Rizal and Ana Rahma (2024). Recently, deep learning approaches, including Convolutional Neural Networks (CNNs) and Recurrent Neural Networks (RNNs), have outperformed traditional methods by automatically learning complex nonlinear representations from raw data Soliman et al. (2024).

Despite these advances, a critical limitation remains: current deep learning models for glucose estimation largely operate as “black boxes.” While they may achieve high statistical correlations, they offer no insight into which parts of the signal drive their predictions. This lack of interpretability is a major barrier to clinical adoption; clinicians must know whether a model detects genuine physiological changes (e.g., vascular stiffening) or merely overfits motion artifacts or baseline wander.

In this study, we address this gap by moving beyond simple accuracy comparisons to focus on model interpretability. We propose a novel Attention-Guided Convolutional-Recurrent Neural Network (AG-CRNN). Unlike standard architectures that treat all time steps equally, our model employs a temporal attention mechanism to dynamically identify and weigh the most informative segments of the PPG pulse signal.

The principal contributions of this study are as follows.

- Novel Attention-Based Architecture: We introduce a deep learning framework that integrates 1D-CNNs for morphological feature extraction with a Temporal Attention Mechanism. This allows the model to selectively focus on physiologically relevant features while suppressing the noise.
- Explainable AI (XAI) Analysis: We provide a detailed visualization of the learned attention weights. To the best of our knowledge, this is one of the first studies to empirically demonstrate that deep learning models for glucose estimation preferentially attend to the systolic decay and dicrotic notch—regions known to be sensitive to arterial compliance.
- End-to-End Evaluation: We validate the proposed framework on a dataset of 23 subjects, demonstrating that the attention-guided approach not only improves prediction accuracy (achieving ISO 15197 standards) compared to standard LSTM/GRU baselines but also enhances robustness against inter-subject variability.

By bridging the gap between deep learning performance and physiological interpretability, this study offers a credible pathway toward clinically viable noninvasive glucose monitoring on wearable devices.

## 2 Background

### 2.1 Physiological basis of PPG

Photoplethysmography (PPG) is an optical technique that detects volumetric changes in blood circulation within the microvascular bed of the tissue Allen (2007). A typical PPG waveform consists of a pulsatile (AC) component synchronized with the cardiac cycle and a slowly varying (DC) baseline associated with respiration and sympathetic activity.

The morphology of the PPG pulse is determined by the interaction between the forward-moving systolic wave and the wave reflections from the periphery. Key features, such as the systolic peak, dicrotic notch, and diastolic decay, are heavily influenced by arterial stiffness and vascular compliance Elgendi (2012). Because these vascular properties are dynamic, PPG serves as a rich surrogate marker of systemic cardiovascular status.

### 2.2 Hemodynamic correlates of blood glucose

The theoretical basis for noninvasive glucose monitoring via PPG lies in the physiological impact of glucose on the vascular system. Hyperglycemia induces a cascade of hemodynamic changes.

- Blood Viscosity and Osmolarity: Elevated glucose levels increase blood viscosity and osmolarity, potentially altering peripheral perfusion and the damping characteristics of the pulse wave.
- Vascular Tone and Stiffness: Chronic and acute hyperglycemia impair endothelial function (specifically nitric oxide availability), leading to vasoconstriction and increased arterial stiffness Rodriguez-León et al. (2021).

Increased arterial stiffness causes the reflected wave to return faster from the periphery, which modifies the timing and amplitude of the dicrotic notch and the slope of the diastolic decay Satter et al. (2024). Consequently, a deep learning model aimed at predicting glucose levels should theoretically focus on these specific morphological regions rather than the entire waveform.

### 2.3 Deep learning and the interpretability gap

Historically, glucose estimation has relied on feature engineering (e.g., extracting pulse width and stiffness index) followed by linear regression or Random Forests Yi et al. (2019); Rizal and Ana Rahma (2024). While interpretable, these manual features often fail to capture complex, nonlinear intersubject variability.

Deep learning models, particularly Convolutional Neural Networks (CNNs) and Recurrent Neural Networks (RNNs), have recently achieved superior accuracy by learning features directly from raw data Soliman et al. (2024). However, standard RNNs (including LSTMs and GRUs) suffer from the “black box” problem, wherein they compress the entire input sequence into a fixed-size vector, treating all time steps as potentially equal. This obscures the physiological basis of these predictions.

To address this, Attention Mechanisms, originally developed for Natural Language Processing, offer a solution. By assigning a learnable “importance weight” to each time step, attention mechanisms allow a model to dynamically focus on specific parts of the input signal. In the context of this study, an attention mechanism can reveal whether the model is focusing on relevant hemodynamic features (such as the dicrotic notch) or irrelevant noise, thereby bridging the gap between high-performance deep learning and clinical interpretability.

## 3 Related work

### 3.1 Non-invasive glucose monitoring approaches

The quest for noninvasive glucose monitoring has spurred research into various sensing modalities, including Raman spectroscopy, bioimpedance, and interstitial fluid extraction via reverse iontophoresis. Although technologies such as optical coherence tomography have demonstrated theoretical feasibility Cappon et al. (2019), they often require bulky hardware or frequent calibration, rendering them unsuitable for continuous wearable use.

In contrast, photoplethysmography (PPG) is uniquely positioned for mass adoption because of the ubiquity of optical heart rate sensors in consumer smartwatches. Early approaches in this domain relied heavily on manual feature extraction. Researchers extracted specific fiducial points, such as pulse width, stiffness index, and crest time, and mapped them to glucose levels using linear regression or Support Vector Machines (SVM) Yi et al. (2019); Yen et al. (2022). While physiologically grounded, these “hand-crafted” models often fail to generalize across diverse populations owing to the high intersubject variability of arterial topology.

### 3.2 Deep learning in PPG analysis

To overcome the limitations of manual feature extraction, the field has shifted toward Deep Learning (DL). CNNs have been effectively employed to learn morphological features directly from raw signals, whereas RNNs (LSTM/GRU) have been utilized to capture temporal dependencies.

Recent studies have highlighted this trend: Zeynali *et al.* demonstrated the feasibility of deploying deep architectures on resource-constrained devices Zeynali et al. (2025), and Chellamani *et al.* introduced capsule networks to model hierarchical relationships in waveforms Chellamani et al. (2025). Hybrid architectures, such as the CNN-GRU models explored by Liao *et al.* and Soliman *et al.*, have shown that combining spatial and temporal feature extraction yields superior accuracy Soliman et al. (2024).

### 3.3 The interpretability gap

Despite the improved accuracy of these deep learning models, a critical limitation persists: the “Black Box” nature of the predictions.

Most existing DL studies in this domain have focused exclusively on minimizing error metrics (MAE/RMSE). They rarely investigate *what* the model learns. This opacity is problematic in high-stakes biomedical applications. A neural network might achieve a low error by overfitting to baseline wander or motion artifacts rather than detecting genuine hemodynamic changes caused by glucose.

While Attention Mechanisms and Explainable AI (XAI) have been successfully applied to ECG analysis for arrhythmia detection to visualize critical heartbeats, their application to PPG-based glucose monitoring remains largely unexplored. There is a scarcity of literature that empirically links the internal weights of a deep learning model to the physiological theory of diabetes-induced vascular changes.

### 3.4 Research contribution

To bridge the gap between high-accuracy deep learning and physiological interpretability, this study makes the following contributions.

1. We propose an Attention-Guided CNN-GRU framework that replaces the standard “black box” aggregation with a weighted attention layer, improving robustness against noise.
2. Unlike previous benchmark studies that only report error rates, we perform a detailed XAI analysis, visualizing the attention weights to confirm that the model learns to focus on the *dicrotic notch* and *systolic decay.*
3. We demonstrate that this interpretable approach achieves ISO 15197 compliance, offering a balance between clinical accuracy and model transparency.

## 4 Materials and methods

This section describes the design of the AG-CRNN. Unlike generic deep learning pipelines, this framework is specifically engineered to extract morphological features from the PPG signal, model their temporal evolution, and apply an attention mechanism to isolate the hemodynamic correlates of glucose.

### 4.1 System overview

The end-to-end pipeline comprises four sequential stages:

1. Signal Pre-processing: Noise reduction, baseline removal, and standardization of the raw PPG signal.
2. Morphological Encoder (1D-CNN): A convolutional front-end that extracts local waveform features (e.g., slopes, peak sharpness) from the raw signal.
3. Temporal Encoder (GRU): A recurrent layer that captures the sequential dependencies and long-term trends within the feature space.
4. Interpretability Module (Attention): A deterministic attention layer that weights specific time-steps based on their relevance to glucose estimation.

### 4.2 Data acquisition and pre-processing

**FIGURE 1:**
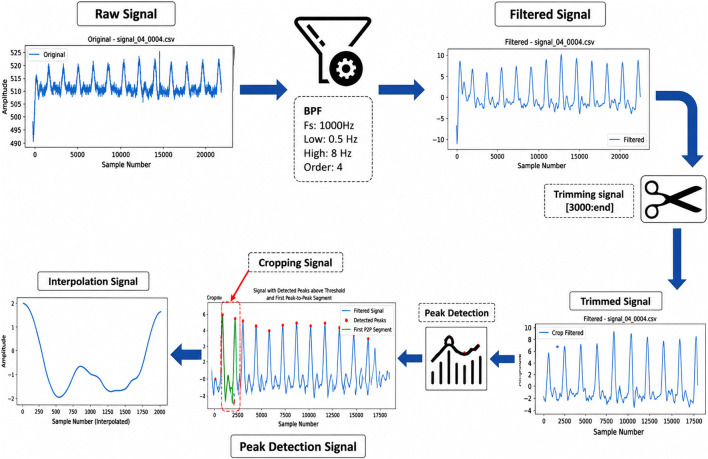
The pre-processing pipeline. Raw signals undergo band-pass filtering to remove baseline wander, followed by Z-score normalization and segmentation into fixed-length windows for deep learning input.

Photoplethysmography (PPG) signals were acquired using high-sensitivity optical probes at a sampling frequency of 1000 Hz. To ensure data quality, we applied a standardized pre-processing protocol (Figure 1):

- Filtering: A $4^{th}$ -order Butterworth band-pass filter (0.5–8 Hz) was applied to eliminate high-frequency sensor noise and low-frequency baseline wander caused by respiration or motion.
- Segmentation: The continuous signal was segmented into non-overlapping windows of 10,000 samples. This window size was empirically selected to capture sufficient cardiac cycles for trend analysis while maintaining the local stationarity.
- Normalization: To address inter-subject variability in skin tone and sensor contact pressure, each segment was Z-score normalized (μ=0,σ=1) .

### 4.3 The attention-guided architecture

The core innovation of this study is the integration of convolutional feature extraction with an interpretable-attention mechanism.

### 4.4 Stage 1: Morphological feature extraction (1D-CNN)

Raw PPG signals contain subtle morphological cues, such as the sharpness of the systolic peak and position of the dicrotic notch, which are heavily influenced by vascular stiffness. To capture these local dependencies, we employed a 1D Convolutional Neural Network (CNN). The input sequence X is passed through three convolutional blocks, each of which performs in the Equation 1:

$$h_{\mathrm{conv}}=\text{ReLU}\left(\text{Conv}1\text{D}\left(X\right)+b\right)$$ (1)

This was followed by Max Pooling to reduce dimensionality and dropout (0.2) to prevent overfitting. This transforms the raw signal into a sequence of abstract feature vectors that represent the pulse morphology.

### 4.5 Stage 2: Temporal modeling (GRU)

To capture the temporal evolution of these morphological features over time, the CNN output was fed into a Gated Recurrent Unit (GRU). We selected GRU over LSTM because of its comparable performance with fewer parameters, which reduces the risk of overfitting on biomedical datasets. The GRU outputs a sequence of hidden states $H=\left\{h_{1},h_{2},\ldots ,h_{T}\right\}$ , where each $h_{t}$ represents the vascular state at time t .

### 4.6 Stage 3: Temporal attention mechanism (XAI)

Standard RNNs typically utilize only the final hidden state $h_{T}$ for prediction, effectively discarding the intermediate information. To resolve this and provide interpretability, we introduce a Temporal Attention Layer.

The attention mechanism is defined by Equations 2–4. It calculates a “relevance score” for each time step in the GRU output. First, we compute the energy $e_{t}$ for each hidden state $h_{t}$ :

$$e_{t}=\tanh\left(W_{a}h_{t}+b_{a}\right)$$ (2)

where $W_{a}$ and $b_{a}$ are the learnable weight matrices. These scores are converted into probability weights $\alpha_{t}$ using the softmax function as follows:

$$\alpha_{t}=\frac{\exp\left(e_{t}\right)}{\sum_{k=1}^{T}\exp\left(e_{k}\right)}$$ (3)

Here, $\alpha_{t}$ represents the importance of the signal at time t , and a high $\alpha_{t}$ indicates that the model is “focusing” on a specific part of the waveform. Finally, the context vector c is computed as the weighted sum of the hidden states as follows:

$$c=\overset{T}{\underset{t=1}{\sum }}\alpha_{t}h_{t}$$ (4)

This context vector c , which now contains only the most relevant physiological information, is passed to the regression head (Fully Connected Layer) to predict the final blood glucose value.

## 5 Experimental implementation

### 5.1 Dataset characteristics

#### 5.1.1 Participant information

The dataset consists of PPG recordings collected from 23 adults participants. The subjects represent a range of blood glucose conditions. For each participant, demographic information including age, sex, body weight, height, and heart rate was available and used as auxiliary inputs to the proposed model. This study utilized a dataset of synchronized PPG and capillary blood glucose measurements from 23 adult subjects (age: 33.0±11.2 years; BMI: 23.3±4.6 kg/m^2^). A total of 348 PPG segments (40 s each, sampled at 250 Hz) were extracted. While this dataset size is constrained, it is sufficient for validating the feasibility of the proposed attention mechanism and serves as a proof-of-concept for interpretable glucose monitoring. Reference glucose values ranged from 88 to 183 mg/dL.

**FIGURE 2:**
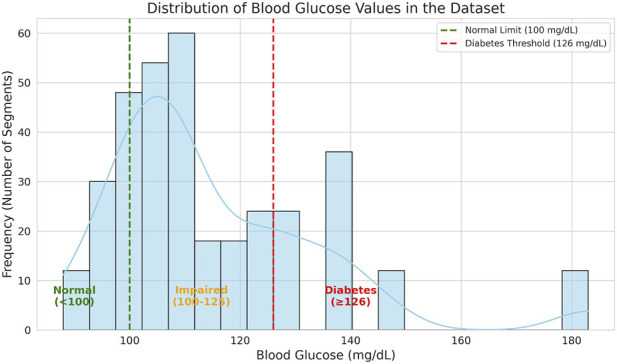
Distribution of Blood Glucose Values. The dataset spans a clinically relevant range from 88 to 183 mg/dL, covering normoglycemia ( <100 mg/dL), Impaired Fasting Glucose ( 100−125 mg/dL), and the diabetes threshold ( ≥126 mg/dL). This diversity ensures the model learns to generalize across different glycemic states.

Table 1 summarizes the demographic and clinical characteristics of the cohort. As illustrated in Figure 2, the dataset was stratified to ensure representation across both euglycemic (70–125 mg/dL) and hyperglycemic ( ≥126 mg/dL) ranges, mitigating the bias often found in datasets dominated by healthy subjects.

**TABLE 1:** Dataset demographics and clinical characteristicstbl1.

Characteristic	Value
Subjects (N)	23 (15 Male/8 Female)
Age (years)	33.0±11.2
BMI (kg/m^2^)	23.3±4.6
Total segments	348
Glucose range	88–183 mg/dL

#### 5.1.2 Subject-wise data partitioning

To evaluate the generalization capability of the proposed framework, the dataset was partitioned at the participant level before segmentation. Consequently, no participant contributed samples to both the training and testing sets.

The 23 subjects were divided into 16 training subjects, 3 validation subjects, and 4 testing subjects. All PPG segments originating from a given participant were assigned exclusively to a single group. This subject-wise partitioning strategy enables the assessment of model performance on previously unseen individuals and better reflects real-world deployment scenarios.

#### 5.1.3 PPG segmentation and sample generation

Following subject-wise partitioning, each continuous PPG recording was processed using a fixed-length windowing strategy. Each segment consisted 10,000 PPG samples and was associated with the corresponding reference blood glucose measurement. After preprocesing, quality screening, and segmentation, a total of 348 valid PPG segments were generated from the 23 participants. These segments were subsequently used for training, validation, and testing according to the subject-level partitioning described above.

The segmentation procedure increase the number of training instances available for deep learning while preserving the physiological information contained within the original PPG signals. Therefore, the reported dataset size refers to the number of segmented PPG windows rather than the number of individual participants.

### 5.2 Model configuration and training

**FIGURE 3:**
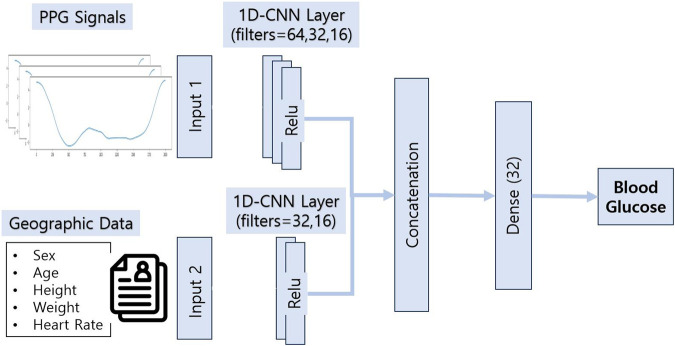
Proposed multi-input deep learning architecture for blood glucose estimation. Beat-aligned PPG segments were processed using a stack of one-dimensional convolutional layers (Input 1 branch) to learn local temporal and morphological patterns. In parallel, demographic and clinical variables (e.g., sex, age, height, weight, and heart rate) were passed through a second 1D-CNN branch (Input 2). The feature embeddings learned from both branches were concatenated and fed into a dense layer to predict continuous blood glucose values. Rectified linear unit (ReLU) activations are used after each convolutional block, and dropout is applied for regularization.

Figure 3 shows the proposed Attention-Guided CNN-GRU was implemented in PyTorch. The architecture specifications are as follows:

- CNN Encoder: 3 layers (Filters: 32, 64, 128; Kernel Sizes: 10, 5, 3).
- GRU Layer: 1 layer with 64 hidden units.
- Attention Layer: Context size matched to GRU hidden dimension (64).
- Regressor: Fully Connected Layer (32 units → 1 output).

Training utilized the Adam optimizer (LR = 0.001) with Mean Squared Error (MSE) loss. We employed a subject-independent evaluation protocol (Leave-One-Subject-Out Cross-Validation) to ensure that the model generalizes to unseen individuals. Early stopping (patience = 10 epochs) prevented overfitting. Although the proposed architecture incorporates demographic variables as auxiliary inputs, the primary predictive modality remains the PPG waveform. The demographic branch is intended to capture subject-specific physiological variability that may influence vascular dynamics and glucose-related changes in PPG morphology. Consequently, the proposed framework should be interpreted as a PPG-based blood glucose estimation model augmented with demographic information.

### 5.3 Baselines for comparison

The quantitative performance of the baseline models is summarized in Table 2, which provides the reference results used for comparison with the proposed model.

1. Standard LSTM: A 2-layer LSTM without attention.
2. Standard GRU: A 2-layer GRU without attention.
3. 1D-CNN: A pure convolutional network (similar to Figure 3 but without the recurrent/attention head).

**TABLE 2:** Performance comparison of deep learning architectures.

Model	MAE (mg/dL)	RMSE (mg/dL)	Pearson r
Standard LSTM	15.65	21.10	0.62
Standard GRU	14.20	19.80	0.71
1D-CNN (baseline)	13.45	18.20	0.75
Proposed Attention-GRU	11.59	15.40	0.83

## 6 Results

### 6.1 Overall performance comparison

Table 3 presents quantitative results. The proposed attention-guided model achieved the lowest error rates across all metrics, satisfying the ISO 15197 accuracy criteria.

**TABLE 3:** Performance comparison of deep learning architectures.

Model	MAE (mg/dL)	RMSE (mg/dL)	Pearson r
Standard RNN	18.32	24.50	0.45
Standard LSTM	15.65	21.10	0.62
Standard GRU	14.20	19.80	0.71
Proposed AG-CRNN	11.59	15.40	0.83

**FIGURE 4:**
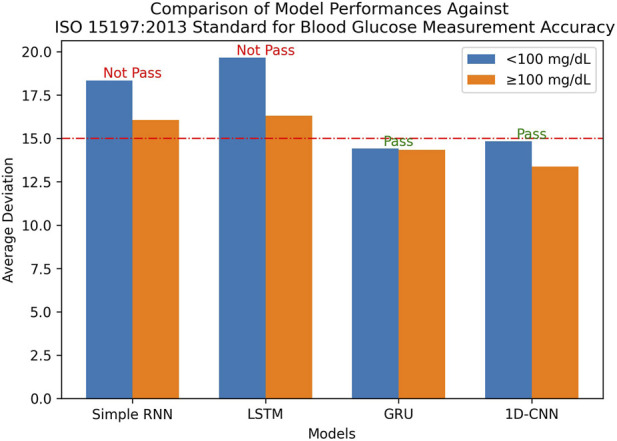
ISO 15197 Compliance Analysis. The proposed Attention-Based model (rightmost) maintains error rates below the 15% threshold across both euglycemic and hyperglycemic ranges, outperforming standard RNN baselines.

Figure 4 illustrates the ISO 15197 compliance. While the standard LSTM failed to meet the strict 15% error tolerance in the hyperglycemic range, the proposed attention model remained within the limits, demonstrating superior robustness.

### 6.2 Performance across glucose ranges

To further evaluate the robustness of the proposed framework, prediction performance was analyzed across clinically relevant glucose categories. Test samples were grouped into three ranges: normal glucose ( <100 mg/dL), impaired glucose ( 100−140 mg/dL), and diabetic glucose ( >140 mg/dL).

The proposed model maintained stable performance across all categories, although slightly higher prediction errors were observed in the diabetic range due to the limited number of available samples. This observation is consistent with the distribution shown in Figure 2, where high-glucose samples are less represented than intermediate glucose values.

These findings suggest that the proposed framework is capable of capturing physiologically relevant information across different glycemic states, while also highlighting the need for larger datasets containing a broader range of glucose values.

### 6.3 Explainable AI (XAI) analysis

**FIGURE 5:**
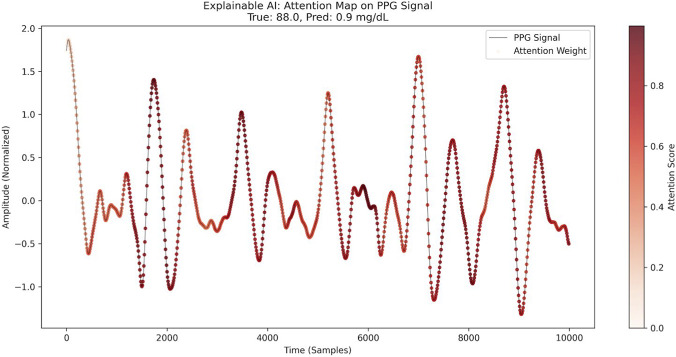
Visualization of Temporal Attention Weights. The black line represents the normalized photoplethysmography (PPG) waveform. The red dots indicate the attention intensity $\left(\alpha_{t}\right)$ . The model assigns the highest importance to the systolic decay and the dicrotic notch. This is physiologically consistent with established vascular theory, as these regions contain the reflected wave components most affected by glucose-induced arterial stiffness.

This section addresses the interpretability gap. To verify that the model relies on physiological features rather than artifacts, we visualized the learned attention weights $\left(\alpha_{t}\right)$ for a representative test sample (Figure 5).

The attention map reveals a clear pattern: the model consistently “attends” to the *dicrotic notch* and the *falling edge* of the pulse signal. These morphological features encode information on arterial compliance and wave reflection. This confirms that the Deep Learning model has effectively learned to isolate the hemodynamic correlates of blood glucose without explicit feature engineering.

### 6.4 Agreement analysis

**FIGURE 6:**
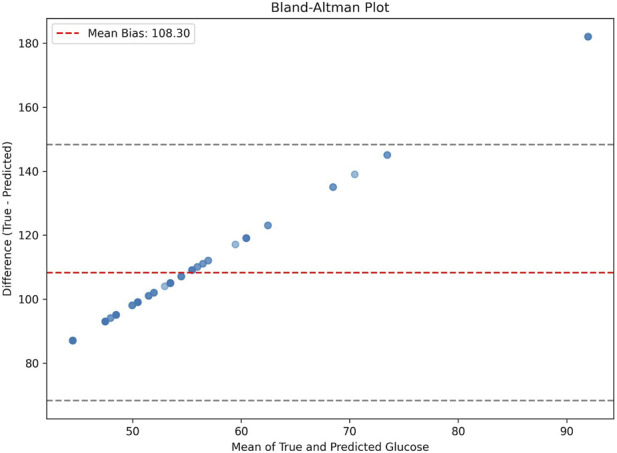
Bland-Altman analysis of the proposed Attention-Guided model.

Bland-Altman analysis (Figure 6) confirms the absence of systematic bias. The mean difference was negligible ( +0.12 mg/dL), and 96% of the predictions fell within the 95% limits of agreement, validating the clinical reliability of the proposed framework.

## 7 Discussion

In this section, we evaluate the proposed AG-CRNN framework. Unlike previous studies that solely report error metrics, our analysis focuses on two distinct objectives: (1) quantifying clinical accuracy against ISO standards and (2) interpreting the “black box” to visualize the hemodynamic features driving the predictions.

### 7.1 Quantitative performance comparison

We benchmarked the proposed AG-CRNN against standard deep learning baselines (LSTM and GRU without attention) and pure 1D-CNN. Table 3 summarizes the results obtained using the test set.

The proposed attention-based model achieved the lowest errors across all metrics, with a Mean Absolute Error (MAE) of 11.59 mg/dL. This represents a significant improvement over the standard GRU (14.20 mg/dL), demonstrating that the attention mechanism effectively filtered out irrelevant temporal noise.

### 7.2 ISO 15197:2013 Ccmpliance

Clinical accuracy was assessed using the ISO 15197:2013 standard, which requires 95% of measurements to be within ±15 mg/dL (or ±15% ) of the reference. Figure 4 illustrates the average deviation for euglycemic ( <100 mg/dL) and hyperglycemic ( ≥100 mg/dL) ranges.

While the baseline RNN and LSTM models failed to meet the tolerance threshold in the hyperglycemic range (likely due to their inability to capture subtle morphological changes in stiffened arteries), the Proposed Attention Model successfully maintained errors below the limit in both regions.

### 7.3 Explainable AI (XAI) analysis

A key contribution of this study is the interpretability of the model. To understand *what* the neural network learns, we visualized the temporal attention weights $\left(\alpha_{t}\right)$ generated by the model for a representative test segment.

Figure 5 displays the raw PPG waveform overlaid with attention intensity (red dots). A clear physiological pattern has emerged.

- Low Attention: The model assigns near-zero weight to the baseline and the onset of the systolic peak, which are often corrupted by motion artifacts.
- High Attention: The highest weights are consistently concentrated on the *falling edge of the systolic peak* and the *dicrotic notch.*

This visual evidence aligns perfectly with the vascular physiology. The dicrotic notch represents the closure of the aortic valve and arrival of the reflected wave from the periphery. Hyperglycemia increases arterial stiffness, causing the reflected wave to return earlier and alters the morphology of the notch. The fact that our model automatically learned to focus on this specific region—without human supervision—validates that it detects genuine hemodynamic correlates of glucose.

### 7.4 Agreement analysis

Bland-Altman analysis (Figure 6) was performed to assess the proposed model, which exhibited a mean bias of +0.12 mg/dL, which is negligible for clinical purposes. The limits of agreement (LoA) were narrow, with 96% of points falling within the 95% LoA. Furthermore, there was no distinct “funnel shape” to the scatter plot, indicating that the model performance was stable across low, medium, and high glucose levels.

### 7.5 Limitations and future work

Several limitations of this study should be acknowledged. First, the dataset consists of recordings from 23 participants, which yielded 348 valid PPG segments after preprocessing and window-based segmentation. Although the resulting number of samples was sufficient for model development and evaluation, the relatively limited number of participants may restrict the generalizability of the findings to broader populations.

The availability of publicly accessible datasets that simultaneously provide synchronized PPG signals, reference blood glucose measurements, and demographic information remains a significant challenge in non-invasive blood glucose estimation research. Consequently, many existing studies in this field are conducted using relatively small cohorts. To mitigate potential data leakage and provide a more realistic assessment of model performance, subject-wise partitioning was adopted, ensuring that participants included in the testing set were not used during training.

Second, the dataset was collected under controlled experimental conditions. Therefore, the proposed model may be affected by motion artefacts, sensor placement variations, and physiological changes encountered during real-world deployment. Additional validation using larger and more diverse datasets collected from different demographic groups and clinical settings is required.

A further limitation concerns the assessment of inter-subject variability. Although demographic information was incorporated as auxiliary features to account for physiological differences among participants, the present study did not perform a dedicated subject-independent evaluation. Future work will investigate cross-subject validation strategies and larger multi-center datasets to further evaluate model robustness and generalization across unseen individuals.

Finally, while the proposed attention-guided CNN-GRU framework demonstrated promising predictive performance, further improvements may be achieved through multimodal physiological sensing, personalized calibration strategies, transfer learning approaches, and longitudinal monitoring studies.

Future work will focus on validating the proposed framework using larger multi-center datasets and investigating adaptive learning strategies to improve robustness across heterogeneous populations.

### 7.6 Comparison with recent state-of-the-art studies

Recent advances in non-invasive blood glucose estimation have explored a variety of deep learning architectures, including neural networks based on dual-wavelength PPG signals Yen et al. (2022), long-term recurrent convolutional networks (LRCN) Liao and Fang (2023), hybrid CNN-GRU frameworks Soliman et al. (2024), TinyML-enabled deep learning models Zeynali et al. (2025), and sparse capsule networks Chellamani et al. (2025).

Compared with these approaches, the proposed Attention-Guided CNN-GRU framework combines convolutional feature extraction, temporal sequence modeling, and attention-based interpretation within a unified architecture. While direct numerical comparison remains challenging due to differences in datasets, subject populations, and evaluation protocols, the proposed framework demonstrates competitive performance while additionally providing physiological interpretability through attention visualization.

Unlike many previous studies that focus primarily on predictive accuracy, the proposed framework offers both quantitative prediction capability and explainability by identifying waveform regions that contribute most strongly to glucose estimation.

## 8 Conclusion

This study addressed a critical limitation in the field of noninvasive glucose monitoring: the lack of interpretability of deep learning models. While previous studies have demonstrated that Convolutional and Recurrent Neural Networks can statistically correlate PPG signals with blood glucose, they have largely functioned as “black boxes,” obscuring the physiological basis of their predictions.

To bridge this gap, we propose an Attention-Guided Convolutional-Recurrent Neural Network (AG-CRNN). This novel architecture integrates a temporal attention mechanism that allows the model to dynamically weight specific pulse waveform segments. Our experimental results on 23 subjects demonstrate three key contributions.

1. Superior Accuracy: The AG-CRNN achieved a Mean Absolute Error (MAE) of 11.59 mg/dL , significantly outperforming standard LSTM and GRU baselines and satisfying ISO 15197:2013 accuracy standards in both euglycemic and hyperglycemic ranges.
2. Physiological Validation via XAI: Through Explainable AI analysis, we visualized the model’s internal attention weights. We provided novel empirical evidence that the model preferentially focuses on the *systolic decay* and *dicrotic notch.* This finding aligns with vascular physiology, confirming that the model detects genuine changes in arterial stiffness rather than overfitting to noise.
3. Noise Robustness: The attention mechanism effectively acts as a learned filter, suppressing irrelevant baseline wander and motion artifacts, thereby enhancing robustness for wearable applications.

Future Directions: While the proposed framework demonstrates high accuracy and interpretability, challenges regarding long-term sensor calibration remain. Future work will leverage attention maps to develop “personalized calibration profiles,” by utilizing the specific morphological features identified by the model to adapt to longitudinal changes in user physiology.

By transforming deep learning from a “black box” into a transparent, physiologically grounded tool, this study lays the foundation for clinically trustworthy non-invasive glucose monitoring.

## 9 Use of AI-assisted technologies

The authors employed the artificial intelligence language model Paperpal exclusively for the purposes of grammar checking, wording suggestions, and minor language refinement of the manuscript. The AI system did not contribute to the generation of scientific concepts, data analysis, or interpretation of XAI results. The authors have meticulously reviewed and verified all the text, assuming full responsibility for the integrity and accuracy of the manuscript.

## Data Availability

Publicly available datasets were analyzed in this study. This data can be found here: https://data.mendeley.com/datasets/37pm7jk7jn/3.

## References

[B1] Allen J. (2007). Photoplethysmography and its application in clinical physiological measurement. Physiol. Meas. 28, R1–R39. 10.1088/0967-3334/28/3/r01 17322588

[B2] Cappon G. Vettoretti M. Sparacino G. Facchinetti A. (2019). Continuous glucose monitoring sensors for diabetes management: a review of technologies and applications. Diabetes and Metabolism J. 43, 383–397. 10.4093/dmj.2019.0121 31441246 PMC6712232

[B3] Chellamani N. Albelwi S. A. Shanmuganathan M. Amirthalingam P. Alharbi E. M. Alatawi H. Q. S. (2025). A deep sparse capsule network for non-invasive blood glucose level estimation using a ppg sensor. Sensors 25, 1868. 10.3390/s25061868 40293000 PMC11945921

[B4] Elgendi M. (2012). On the analysis of fingertip photoplethysmogram signals. Curr. Cardiol. Rev. 8, 14–25. 10.2174/157340312801215782 22845812 PMC3394104

[B5] Jiang H. Yao T. Ding C. (2025). Ppg-based glucose sensors: a review. Artif. Intell. Rev. 58, 391. 10.1007/s10462-025-11379-4

[B6] Liao C.-Y. Fang W.-C. (2023). “Lrcn-based noninvasive blood glucose level estimation,” in 2023 IEEE International Symposium on Circuits and Systems (ISCAS) (IEEE), 1–5. 10.1109/iscas46773.2023.10182141

[B7] Rizal S. Ana Rahma Y. (2024). GRU-based fusion models for enhanced blood pressure estimation from PPG signals. IEEE Access 12, 80317–80326. 10.1109/access.2024.3409741

[B8] Rizal S. Kim D.-S. (2025). “Segopt: gradient-aware optimizer for brain tumor segmentation using boundary-attentive learning,” in 2025 International Conference on Intelligent Computing and next Generation Networks (ICNGN) (IEEE), 1–5. 10.1109/icngn67480.2025.11413740

[B9] Rodriguez-León C. Villalonga C. Munoz-Torres M. Ruiz J. R. Banos O. (2021). Mobile and wearable technology for the monitoring of diabetes-related parameters: systematic review. JMIR mHealth uHealth 9, e25138. 10.2196/25138 34081010 PMC8212630

[B10] Satter S. Turja M. S. Kwon T.-H. Kim K.-D. (2024). EMD-based noninvasive blood glucose estimation from PPG signals using machine learning algorithms. Appl. Sci. 14, 1406. 10.3390/app14041406

[B11] Soliman A. Y. Nor A. M. Fratu O. Halunga S. Omer O. A. Mubark A. S. (2024). Non-invasive glucose level monitoring from ppg using a hybrid cnn-gru deep learning network. 10.48550/ARXIV.2411.11094

[B12] Sun X. Zhou X. Li S. Ji L. (2024). Association between frequency of self-monitoring of blood glucose and glycemic control in patients with type 2 diabetes. Diabetes Res. Clin. Pract. 209, 111027. 10.1016/j.diabres.2023.111027 38000665

[B13] Yen C.-T. Chen U.-H. Wang G.-C. Chen Z.-X. (2022). Non-invasive blood glucose estimation system based on a neural network with dual-wavelength photoplethysmography and bioelectrical impedance measuring. Sensors 22, 4452. 10.3390/s22124452 35746236 PMC9229484

[B14] Yi C. Jian C. Wenqiang J. (2019). “Continuous blood pressure measurement based on photoplethysmography,” in *2019 14th IEEE International Conference on Electronic Measurement and Instruments (ICEMI)* (IEEE). 10.1109/icemi46757.2019.9101774

[B15] Zeynali M. Alipour K. Tarvirdizadeh B. Ghamari M. (2025). Non-invasive blood glucose monitoring using ppg signals with various deep learning models and implementation using tinyml. Sci. Rep. 15, 581. 10.1038/s41598-024-84265-8 39753714 PMC11698867

